# Synthesis and Characterization of Cu_2_ZnSnS_4_ Thin Films Obtained by Combined Magnetron Sputtering and Pulsed Laser Deposition

**DOI:** 10.3390/nano11092403

**Published:** 2021-09-15

**Authors:** Mohamed-Yassine Zaki, Florinel Sava, Angel-Theodor Buruiana, Iosif-Daniel Simandan, Nicu Becherescu, Aurelian-Catalin Galca, Claudia Mihai, Alin Velea

**Affiliations:** 1 National Institute of Materials Physics, Atomistilor 405A, 077125 Magurele, Romania; yassine.zaki@infim.ro (M.-Y.Z.); fsava@infim.ro (F.S.); angel.buruiana@infim.ro (A.-T.B.); simandan@infim.ro (I.-D.S.); ac_galca@infim.ro (A.-C.G.); claudia.mihai@infim.ro (C.M.); 2 Faculty of Physics, University of Bucharest, 405 Atomiștilor Street, P.O. Box MG-11, 077125 Bucharest-Magurele, Romania; 3 Apel Laser Ltd., Vanatorilor 25, 077135 Mogosoaia, Romania; becherescu@gmail.com

**Keywords:** CZTS, PLD, magnetron sputtering, hybrid system, solar cells

## Abstract

Cu_2_ZnSnS_4_ (CZTS) is a complex quaternary material, and obtaining a single-phase CZTS with no secondary phases is known to be challenging and dependent on the production technique. This work involves the synthesis and characterization of CZTS absorber layers for solar cells. Thin films were deposited on Si and glass substrates by a combined magnetron sputtering (MS) and pulsed laser deposition (PLD) hybrid system, followed by annealing without and with sulfur powder at 500 °C under argon (Ar) flow. Three different Cu_2_S, SnS_2_, and ZnS targets were used each time, employing a different target for PLD and the two others for MS. The effect of the different target arrangements and the role of annealing and/or sulfurization treatment were investigated. The characterization of the absorber films was performed by grazing incidence X-ray diffraction (GIXRD), X-ray reflectometry (XRR), Raman spectroscopy, scanning electron microscopy, and regular transmission spectroscopy. The film with ZnS deposited by PLD and SnS_2_ and Cu_2_S by MS was found to be the best for obtaining a single CZTS phase, with uniform surface morphology, a nearly stoichiometric composition, and an optimal band gap of 1.40 eV. These results show that a new method that combines the advantages of both MS and PLD techniques was successfully used to obtain single-phase Cu_2_ZnSnS_4_ films for solar cell applications.

## 1. Introduction

Cu_2_ZnSnS_4_ (CZTS) is a strong material candidate for next-generation solar energy converters due to its high absorption coefficient and optimal band gap [1]. P-type chalcogenides are made of low-cost and earth-abundant elements (Cu, Zn, Sn, and S), unlike other thin-film solar cell technologies (CIS, CIGS, and CdTe) that contain expensive and/or toxic components [2]. Several physical and chemical routes can be used for the synthesis of CZTS thin films. Although vacuum techniques can be complex and require high energy, they are collectively regarded as a solution to overcome issues related to time consumption, cracks, and poor crystallinity of more used chemical routes [3,4]. They also offer the possibility to easily control the structural and morphological properties of the thin films [5].

Among all physical vapor deposition methods, magnetron sputtering (MS) and pulsed laser deposition (PLD) have largely been used in the preparation of CZTS absorber layers because of their numerous advantages [6,7]. PLD is a simple and versatile technique that is commonly used for depositing thin films of a very wide range of materials on a vast variety of substrates [8,9]. This technique is known for its high deposition rate, relatively easy transfer of species from the target to the substrate, and high-quality growth of thin films due to the use of high energy [10,11]. It offers the possibility to synthesize CZTS films in one step, using a CZTS target and heating the substrates at high temperatures or in two steps by the deposition of the metallic (Cu, Zn, and Sn) film, followed by sulfurization in an enclosed chamber, where the sulfurization or H_2_S vapors is carried out by a flow of argon or nitrogen at the heated surface of the thin film. Various parameters can influence the properties of the films such as the distance between target and substrate, orientation and temperature of the substrate, laser energy, and target composition. Metallic Cu, Zn, and Sn targets can be used to control layer thickness and roughness [5], and a single CZTS target made from CuS, ZnS, SnS, and other binary sulfides is preferred when the goal is to obtain perfectly stoichiometric Cu_2_ZnSnS_4_ materials [12]. Although PLD has great advantages, there are some limitations that hinder the development and research on CZTS-based solar cells using this technique. A major problem is the high number of droplets formed in the films, leading to the apparition of unwanted phases and inhomogeneity [13] (when the single target is made from multiple crystalline phases, or when several targets are used). Other disadvantages are the long time needed to obtain films with large thicknesses [14] and the difficulty to produce large-area films.

Magnetron sputtering is known as a physical technique for the synthesis of highly uniform thin films on large-area substrates [15]. In the MS technique, different parameters (pressure, power, deposition time, etc.) can be varied in order to determine the optimal conditions to obtain high-quality films. In the case of CZTS preparation, MS allows two different approaches, either by substrate heating during deposition or by sulfurization after sputtering deposition [16,17]. The synthesis of CZTS thin films can be performed using different targets, such as metallic precursors, binary sulfide targets, or single quaternary targets [18]. For elemental electrically conductive targets (Cu, Zn, and Sn), it is preferable to use direct current (DC) sputtering [19]. For binary and quaternary targets such as ZnS, Sn_x_S_y_, Cu_x_S, and CZTS, radio frequency (RF) is commonly used [20,21]. The MS method offers the possibility to control the chemical composition and the film thickness to obtain good uniformity on the surface of the sample [22]. MS provides high adhesion of the films and excellent coverage of the substrate surface [23]. High deposition rates and high-purity films can be achieved using MS [24]. However, some issues in MS are related to target poisoning by reactive gas and target cracking at high power [25]. Additionally, with a damaged target, the sputter rate can change significantly, and it can be difficult to control the quantity of the elements incorporated into the film [26].

In order to obtain the advantages of the two techniques mentioned above, and to avoid some of their drawbacks, combining the two methods is a promising route. Benetti et al. studied the deposition of TiO_2_ thin films using a combination of PLD and MS [27]. They indicated that this approach can be very useful in thin-film synthesis, since it provides a high deposition rate compared to the use of either a single PLD or MS system. Additionally, the synthesis of a single-phase quaternary material such as CZTS can be very challenging due to the difficulty of the stoichiometry control and the omnipresence of binary and ternary secondary phases such as ZnS, Cu_x_S, Sn_x_S_y_, and Cu_x_SnS_y_ (CTS) [28,29]. Combining PLD with MS in a hybrid co-deposition process can be beneficial to obtain CZTS films with the desired properties.

In that view, here, we employed a hybrid deposition system (abbreviated MSPLD), where MS and PLD were used simultaneously for CZTS thin-film co-deposition. Three different targets (two for MS and one for PLD), copper sulfide (Cu_2_S), tin sulfide (SnS_2_), and zinc sulfide (ZnS), were used in a simultaneous co-deposition MSPLD process. The system was configured and adjusted during the realization of depositions, as described in a previous study [30], in order to function optimally for each material. The effect of heat treatment with and without sulfurization on the properties of the CZTS thin films was also investigated.

## 2. Materials and Methods

CZTS thin films were obtained using three binary sulfide targets (Cu_2_S, SnS_2_, and ZnS) with a diameter of 2 inches and a thickness of 3 mm in a hybrid system combining MS and PLD techniques. Two targets were mounted in cathodic sprayers (magnetrons), and one in the PLD carousel, and the thin films were synthesized on silicate glass and Si/SiO_2_ substrates. Three CZTS films were deposited at room temperature on each type of substrate by fixing a target to be deposited by PLD and the two others sputtered by MS, all deposits taking place simultaneously. A COMPexPro KrF laser source (Coherent, California, USA) with a wavelength λ of 248 nm and a fluence of 1.5 J/cm^2^ was focused on the target at an incidence angle of 45°. The sputtering was performed using argon as the working gas with a 5 × 10^−3^ Torr (Linde, Bucharest, Romania). The CZTS films were deposited using a Model AG 0313 source (RF T&C Power Conversion Inc., New York, NY, USA) and a 3G Circular Magnetron (Gencoa, Liverpool, UK). In order to define the deposition rate, calibration was performed on the three targets using Q-pod software (Inficon, Bad Ragaz, Switzerland) connected to a quartz crystal. The distance target to sample was 8 cm and 11 cm for the PLD and MS targets, respectively, and the substrate holder was rotated to achieve a uniform deposition.

The first target arrangement utilized a Cu_2_S target for PLD deposition and SnS_2_ and ZnS targets for MS. The PLD parameters for the Cu_2_S layer were 10 Hz for the laser pulse frequency and 210 mJ for the power. The deposition rate was 0.50 Å/s. The SnS_2_ target was deposited using an RF power supply with a power of 20 W to obtain a deposition rate of 0.49 Å/s. The ZnS target was ignited by the RF power supply, and a power of 67 W was applied, giving a deposition rate of 0.50 Å/s.

In the second arrangement, the SnS_2_ target was loaded in the PLD carousel, and the Cu_2_S and ZnS targets were mounted in the magnetrons. The laser pulse frequency was 1 Hz, and the power was maintained at 90 mJ, giving a deposition rate of 0.47 Å/s. The Cu_2_S and ZnS targets were deposited using an RF power supply with a power of 27 W for Cu_2_S and 65 W for ZnS, while the deposition rate was 0.54 Å/s and 0.47 Å/s for the Cu_2_S and ZnS targets, respectively.

In the third arrangement, deposition was performed by PLD from the ZnS target, while MS was used for Cu_2_S and SnS_2_. PLD deposition was carried out using a laser frequency of 2 Hz and a power of 60 mJ. The deposition rate was about 1.30 Å/s. An RF power supply with a power of 56 W to maintain a rate of 1.30 Å/s was applied in the case of the Cu_2_S MS deposition. For the SnS_2_ target, an RF power supply with a power of 40 W was used to obtain a deposition rate of 1.30 Å/s.

The as-deposited samples were named depending on the target deposited by the PLD technique: Cu_2_S (PLD), SnS_2_ (PLD), and ZnS (PLD). Two series of samples were prepared, and each was annealed in an oxygen-free atmosphere inside a quartz tube (2 inches in diameter) of a tubular furnace to form the CZTS phase. The first series, deposited on Si/SiO_2_ substrates, was annealed without any sulfur source and denoted Cu_2_S (PLD)/annealed, SnS_2_ (PLD)/annealed, and ZnS (PLD)/annealed. The second series, deposited on glass substrates, was sulfurized using 1 g of S powder. The samples were named Cu_2_S (PLD)/sulfurized, SnS_2_ (PLD)/sulfurized, and ZnS (PLD)/sulfurized. The two series of thin films were annealed in Ar gas atmosphere at 500 °C for 3 h using a GSL 1600X furnace (MTI, California, USA) with an increment of 2.5 °C/min for heating and cooling.

Grazing incidence X-ray diffraction (GIXRD) with an incidence angle of 0.3° was used to inspect the structural properties of the CZTS thin films. The measurements were performed with a Rigaku SmartLab diffractometer provided with Cu Kα radiation (λ = 1.54178 Å) and HyPix-3000 2D Hybrid Pixel Array Detector (Rigaku, Tokyo, Japan) (in 0 D mode). The X-ray reflectometry (XRR) data were recorded with the same equipment in a range of (0 ÷ 2) (° 2θ), with a 2θ step of 0.004°.

The Raman spectrometer used was LabRAM HR Evolution from HORIBA Jobin-Yvon equipped with a confocal microscope and a He−Ne laser and employed to confirm the crystalline structure study. The measurements were performed with a red excitation wavelength of 633 nm, and the laser was focused using an Olympus 100× objective on the surface of the samples. Raman spectra were analyzed in the range of 200 to 500 cm^−1^ at room temperature.

The optical properties were measured using a V-VASE Woollam Spectroscopic Ellipsometer equipped with a high-pressure Xenon discharge lamp incorporated in an HS-190 monochromator.

The microstructure of the films was examined using a Zeiss EVO 50 XVP scanning electron microscope (SEM) accessorized with an energy-dispersive spectrometer (EDS) for the determination of the elemental concentration in the films.

## 3. Results and Discussion

**Figure 1:**
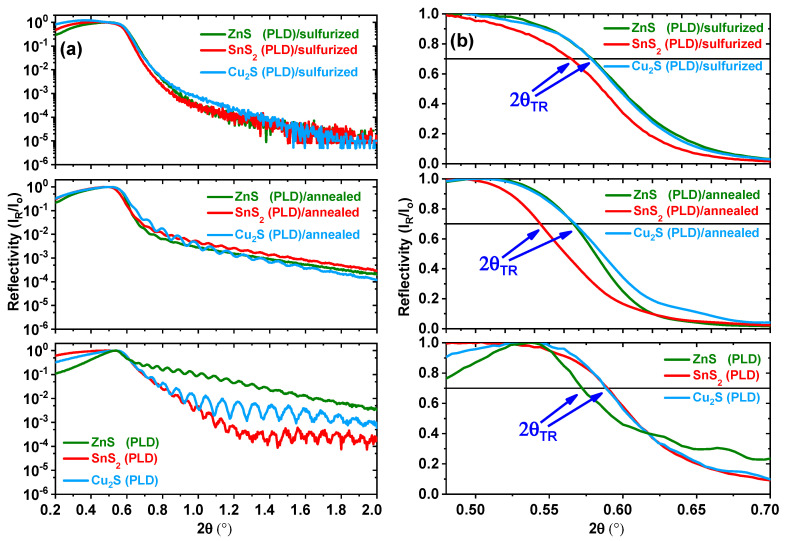
(**a**) XRR diagrams for co-deposited CZTS thin films in the as-deposited state, after annealing at 500 °C and after sulfurization at 500 °C. (**b**) Positions of the total reflection edge (2θ_TR_), defined as the angle for which the reflectivity decreases by 30% of the maximum.

From the XRR diagrams of the three thin films obtained by MSPLD co-deposition, in the initial state and after annealing and sulfurization at 500 °C (Figure 1a), the positions of the total reflection edge (2θ_TR_) (Figure 1b) and the average film thickness are calculated and presented in Table 1. The total reflection edge is defined as the angle for which the reflectivity decreases by 30% of the maximum. We notice that, after the heat treatment without sulfur, 2θ_TR_ decreases slightly for all the films. The average mass density (ρ_m_) of the thin films is proportional with (θ_TR_)^2^, and accordingly, ρ_m_ also decreases (Table 1). This fact, together with the decrease in the average thickness of all annealed CZTS thin films, means that the thin films undergo a process of partial evaporation and a reorganization of atoms, which produces the formation of voids between crystallites and a decrease in the thin-film thickness. The ZnS (PLD) thin film seems to be the least influenced by annealing without sulfur (both ρ_m_ and h_m_ decrease with −2.1% and −2.0%, respectively), the mass loss being only −4.1%. SnS_2_ (PLD) is the most affected (both ρ_m_ and h_m_ decrease with −14.7% and −14.3%, respectively), the mass loss being −29.0%. This might be due to the evaporation of the very volatile SnS [31] during the deposition and annealing. The Cu_2_S (PLD) thin film suffers moderate modifications (both ρ_m_ and h_m_ decrease with −7.0% and −10.2%, respectively), the mass loss being −17.2%. The ZnS (PLD) sample is the densest and shows the smallest modifications in mass density and thickness after annealing. This could be due to the fact that in this case the deposition rate was double in comparison to the other two samples and the deposition rate influences the growth of the film (smoother films with smaller particle sizes are obtained at higher rates). When annealing with sulfur, the XRR fringes are not visible, meaning that the surface of the film becomes rough (confirmed by SEM), which is beneficial for solar cells. However, in this case, we expect a slight increase in the film thickness [32]. Additionally, the decrease in ρ_m_ is much diminished for Cu_2_S (PLD)/sulfurized and SnS_2_ (PLD)/sulfurized, while for ZnS (PLD)/sulfurized, ρ_m_ slightly increases, which means that the voids between crystallites are smaller than in the case of simple annealing.

**Figure 2:**
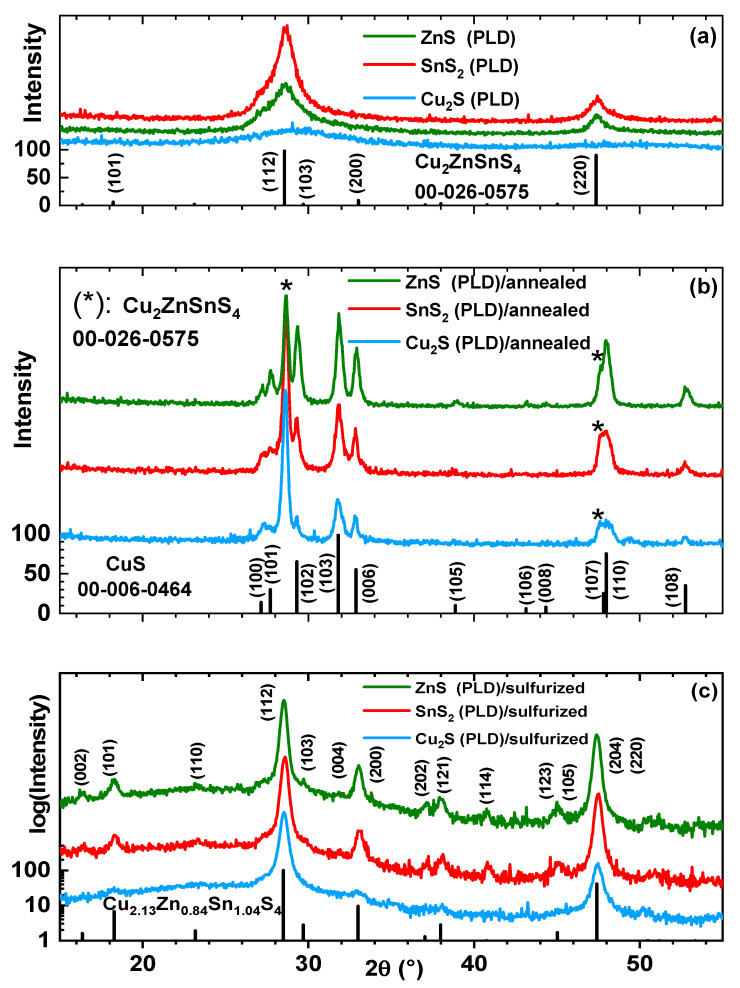
Grazing incidence X-Ray diffraction patterns of the MSPLD thin films (**a**) as-deposited, (**b**) annealed at 500 °C, and (**c**) sulfurized at 500 °C.

Figure 2 shows the grazing incidence X-ray diffraction patterns of the CZTS thin films prepared using the MSPLD hybrid system by switching the target in the PLD carousel in each film and annealing without and with sulfur powder. The GIXRD diagrams for the as-deposited CZTS thin films are presented in Figure 2a. The Cu_2_S (PLD) sample has an amorphous structure, while for the SnS_2_ (PLD) and ZnS (PLD) samples, the amorphous phase is only 12% and 27%, respectively. The majority phase for the last two samples is polycrystalline Cu_2_ZnSnS_4_ (tetragonal, I-42m (121), as natural mineral “stannite”, ICDD file 00-026-0575) with an average size of crystallites of several nanometers. In order to prove the beneficial effect of sulfurization, we also annealed a set of samples without using sulfur. In this case (Figure 2b), all the samples suffer a major transformation: the amorphous phase and the Cu_2_ZnSnS_4_ nanophase (in the case of ZnS (PLD)/annealed and SnS_2_ (PLD)/annealed) are decomposed, some of the Zn and Sn atoms evaporate (thus the concentration of Cu atoms increase in thin films), the sulfur atoms diffuse in the entire volume of the thin films or evaporate (as XRR revealed), and thus, a polycrystalline CuS phase (hexagonal, P63/mmc (194), ICDD file 00-006-0464) appears. The average size of the crystallites for this phase is of hundreds of nanometers. Additionally, the average size of the crystallites for the remaining Cu_2_ZnSnS_4_ phase (tetragonal, I-42m (121), ICDD file 00-026-0575) increases to hundreds of nanometers. In Figure 2c are represented the GIXRD diagrams for the samples sulfurized at 500 °C. All the samples consist of a single phase, Cu_2.13_Zn_0.84_Sn_1.04_S_4_, with the tetragonal signature, the atomic plane (101), and some disorder distribution of the cations in the lattice (space group I-4 (82), similar to natural mineral “kesterite”, ICDD file 04-023-6314). The ZnS (PLD)/sulfurized sample appears to have sharper peaks than the other samples, suggesting an enhancement in crystallinity and particle growth after sulfurization [33]. Therefore, to confirm the suggested improvement, the average crystallite size D of the three sulfurized samples was calculated from the (112) peak of GIXRD data using the Scherrer formula: D = kλ/(β.cosθ), where D is the average crystallite size, k is the Scherrer constant, λ is the wavelength of Cu Kα, β is the corrected FWHM (by eliminating instrumental broadening) of the (112) peak, and θ is the Bragg angle. The D values are 69.40 nm for Cu_2_S (PLD)/sulfurized, 114.50 nm for SnS_2_ (PLD)/sulfurized, and the highest value of 153.24 nm for the ZnS (PLD)/sulfurized sample.

**Figure 3:**
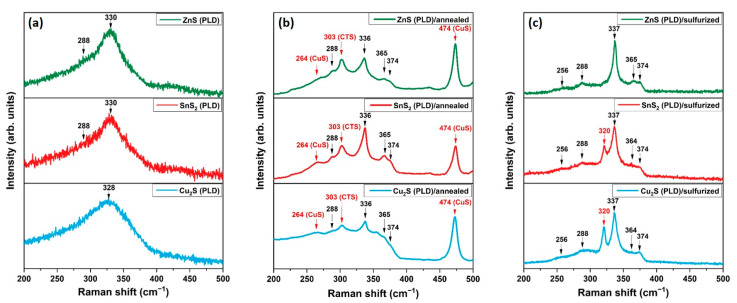
Raman spectra of the MSPLD samples (**a**) as-deposited, (**b**) annealed without sulfur at 500 °C, and (**c**) sulfurized at 500 °C.

In order to confirm the results obtained by GIXRD, and to steadily identify the secondary phases in our samples, Raman spectroscopy was performed with an excitation wavelength of 633 nm. The effect of annealing without and with sulfur powder on the as-deposited thin films was studied and is presented in Figure 3. Two broad peaks at 288 cm^−1^ and 330 cm^−1^ are seen in all the as-deposited samples (Figure 3a). The main Raman peak of the kesterite CZTS phase is located at 336–338 cm^−1^ [34] but was reported to shift to lower values due to some disorder distribution of the cations in the lattice of the CZTS film [35]. Thus, these two peaks are identified as the characteristic peaks of the kesterite CZTS phase. Figure 3b shows the Raman spectra of the samples annealed without sulfur. CZTS peaks can be noticed in the three films at 288, 336, 365, and 374 cm^−1^ [36,37]; however, all the samples contain a strong peak at 474 cm^−1^ and a smaller one at 264 cm^−1^, indicating the presence of CuS [33]. In addition, a third phase is also detected in the films at 303 cm^−1^ and can be associated with the CTS phase [38]. The sulfurized CZTS thin films are presented in Figure 3c. Both the Cu_2_S (PLD)/sulfurized and SnS_2_ (PLD)/sulfurized samples are characterized by five peaks at 256, 288, 337, 364, and 374 cm^−1^ of the CZTS phase and a prominent peak at 320 cm^−1^ whose origin is unclear. Several researchers attributed this peak to the CTS [39,40] or SnS_2_ [41,42] secondary phases, while others stated that it belongs to CZTS [43,44]. On the other hand, the ZnS (PLD)/sulfurized sample contains peaks located at 256, 288, 337, 365, and 374 cm^−1^, which are uniquely characteristic of the CZTS phase [43,45], and no undesirable secondary phase was observed. As a side note, Raman measurements were also performed on all the samples using an excitation wavelength of 325 nm to detect the zinc sulfide phase [46], and no evidence of the ZnS or SnS_2_ secondary phases was detected. The annealing treatment either with or without sulfur plays a considerable role in the formation and crystallization of the CZTS films. However, a simple heat treatment without any sulfur source can lead to the apparition of several secondary phases, no matter what target was used for PLD, most probably due to the lack of sulfur and the very likely evaporation of zinc and tin in the form of SnS, resulting from the decomposition of SnS_2_ at high temperature [47]. As an alternative, sulfurization is primordial for synthesizing single-phase CZTS thin films, as observed in the ZnS (PLD)/sulfurized sample.

**Figure 4:**
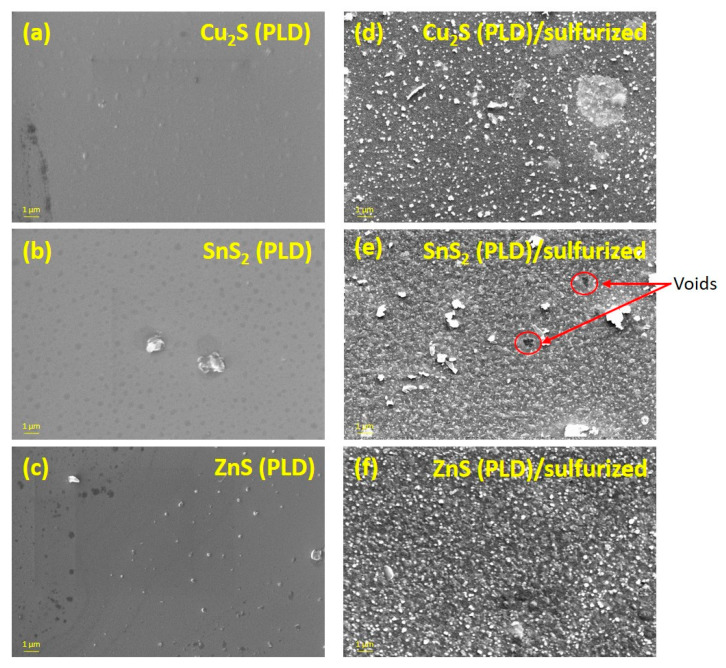
Surface SEM images of the samples on glass substrate as-deposited and sulfurized: (**a**) Cu_2_S (PLD), (**b**) SnS_2_ (PLD), and (**c**) ZnS (PLD) (as-deposited); (**d**) Cu_2_S (PLD)/sulfurized, (**e**) SnS_2_ (PLD)/sulfurized, and (**f**) ZnS (PLD)/sulfurized (sulfurized).

Figure 4 shows the SEM images of the as-deposited and sulfurized CZTS thin films. The surface of the as-deposited films (Figure 2a–c) is smooth, and no particles are observed. On the other hand, the surface of the films sulfurized at 500 °C (Figure 4d–f) is very rough, and agglomerations of hundreds of nanometers are visible. The Cu_2_S (PLD)/sulfurized sample (Figure 4d) is composed of a mixture of small and big particles. In Figure 4e, the SnS_2_ (PLD)/sulfurized sample exhibits a distribution of particles with different shapes and sizes along with some voids probably due to the evaporation of tin sulfide. The ZnS (PLD)/sulfurized film (Figure 4f) shows a more homogeneous and compact surface with monodispersed particles and no voids or cracks. The difference in particles sizes and shapes in the Cu_2_S (PLD)/sulfurized and SnS_2_ (PLD)/sulfurized samples can be explained by the co-existence of secondary phases [48], while a homogeneous surface with relatively monodispersed particles (ZnS (PLD)/sulfurized sample) suggests the existence of a single-phase compound, which further confirms the GIXRD and Raman results.

**Figure 5:**
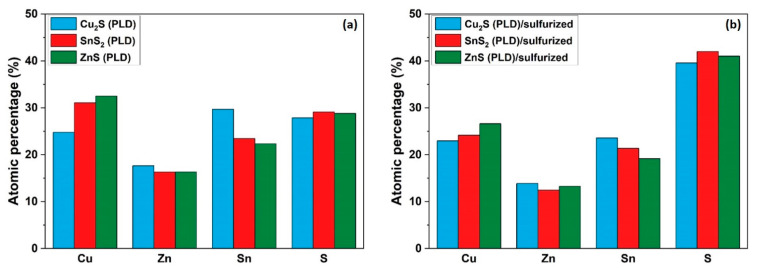
Average atomic ratios of Cu, Zn, Sn and S in the samples obtained in the hybrid MSPLD deposition system: (**a**) as-deposited and (**b**) sulfurized.

An estimation of the average atomic ratios of the four elements (Cu, Zn, Sn, and S), obtained from the energy-dispersive spectroscopy (EDS) analysis, in the as-deposited and sulfurized samples, is presented in Figure 5. All the as-prepared samples exhibit a Sn-rich and S-poor composition compared to the stoichiometric Cu_2_ZnSnS_4_ (Cu_0.25_Zn_0.125_Sn_0.125_S_0.5_) phase. By sulfurization at 500 °C, an improvement in the elemental composition is clearly seen in all the samples (Figure 5b), since the amounts of Cu, Zn, and Sn decreased, and the S quantity increased. However, the sulfurized samples are still off-stoichiometric, rich in tin, and slightly poor in sulfur. The existence of tin in excess can cause the formation of the SnS or SnS_2_ secondary phases [49]. This explains the apparition of the peak at 320 cm^−1^ in both samples (Cu_2_S (PLD)/sulfurized and SnS_2_ (PLD)/sulfurized) in the Raman analysis, which can be definitely attributed to the SnS_2_ phase. One should note that the determination of elemental concentrations by EDS can be affected by several parameters such as the sample thickness and the scattering power of atoms; therefore, significant errors are expected from this characterization technique [48].

**Figure 6:**
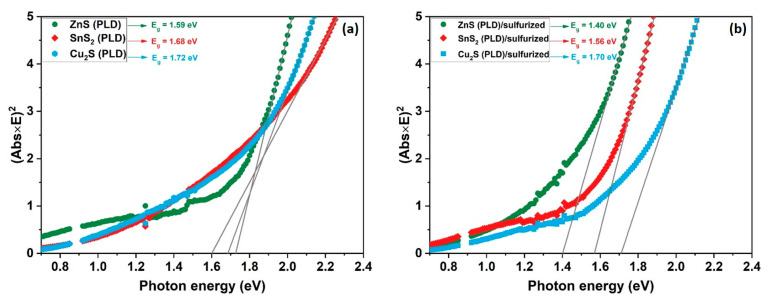
Tauc plot of the (**a**) as-deposited and (**b**) sulfurized CZTS thin films.

The band gaps of the as-deposited and sulfurized CZTS thin films are shown in Figure 6. The transmittance measurements were performed using an ellipsometer, and the optical band gap was determined using the following equation [50]:

Abs(E) = ln (1/T) = C.d.√(E − E_g_) (1)

where Abs(E) is the derived absorbance using the measured transmittance (T), E is the photon energy, C is the constant of proportionality, d is the film thickness, and E_g_ is the optical band gap. E_g_ was estimated using the Tauc plot by plotting (αE)^2^ vs. (E) (where α is the absorption coefficient) near the absorption edge. Since the as-deposited samples in Figure 6a are mostly composed of an amorphous structure, the values of the optical band gaps are above the reported value for the CZTS thin films (≈1.5 eV). In Figure 6b are shown the band gaps of the CZTS sulfurized films. The estimated E_g_ is around 1.40, 1.56, and 1.70 eV for ZnS (PLD)/sulfurized, SnS_2_ (PLD)/sulfurized, and Cu_2_S (PLD)/sulfurized, respectively. The band gap is found to be correlated with the stoichiometry and the elemental composition of the CZTS material. Large E_g_ are caused by an increase in Sn content according to Malerba et al. [51], while Hamanaka et al. reported that the large values could be due to high Zn ratios along with a decrease in Cu content [52]. E_g_ also depends on the content of the three elements, more precisely on the Cu/(Zn + Sn) ratio, since higher energies are obtained with lower values of this ratio and vice versa [53]. This is confirmed in our case, as the Cu/(Zn + Sn) ratio in the Cu_2_S (PLD)/sulfurized sample is very small, and thus, a high value of the optical band gap (1.70 eV) is found. On the other hand, the ZnS (PLD)/sulfurized sample shows an enhancement in this ratio, leading to a lower band gap (1.40 eV). The optical properties of the CZTS thin films obtained here are in good agreement with the literature and are suitable for use in solar cells as absorber layers [54,55].

From the structural, morphological, compositional, and optical results, it can be deduced that the MSPLD hybrid system can be beneficial to the formation of the CZTS kesterite phase. This approach gives the possibility to produce rough films with larger crystallites when compared to some CZTS films prepared either by PLD [11] from a single Cu_2_ZnSnS_4_ target or by MS [17] from a quaternary CZTS alloy target. The XRD and Raman spectroscopy characterizations of the sulfurized films show the formation of a single CZTS crystalline phase in one set of samples (ZnS (PLD)/sulfurized) or the presence of one secondary phase (SnS_2_) in the other samples (SnS_2_ (PLD)/sulfurized and Cu_2_S (PLD)/sulfurized). On the other hand, CZTS absorber layers elaborated by MS can contain several secondary phases [21,56,57] such as Cu_2_S, CTS, and ZnS when multiple binary chalcogenide targets are used or even when a single alloyed CZTS target is sputtered, which can have a harmful impact on the performance of CZTS-based solar cells [58,59]. Additionally, one of the major drawbacks of the PLD method is the use of tiny substrates [60]. The problem can be solved and large-area substrates can be covered by implementing a hybrid MSPLD system that still benefits from PLD advantages. This can be helpful for large-scale industrial CZTS elaboration.

## 4. Conclusions

CZTS thin films were co-deposited simultaneously using three targets (Cu_2_S, SnS_2_, and ZnS) in a hybrid synthesis system combining magnetron sputtering (for two targets) and pulsed laser deposition (for the third target). The effect of the heat treatment with and without a sulfur atmosphere on the material properties was studied. The results show that a simple heat treatment without any sulfur source leads to the evaporation of Zn and Sn and the apparition of CuS and CTS secondary phases in all the films, no matter the target arrangement used. On the other hand, the sulfurized samples show that annealing in a sulfur atmosphere is an essential process for the formation of the CZTS phase. It is found that the samples obtained by simultaneous deposition of ZnS by PLD, Cu_2_S, and SnS_2_ by MS and sulfurized at 500 °C, which leads to the formation of a single-phase CZTS, have the best structural, compositional, and optical properties, confirmed by GIXRD, Raman, SEM-EDX, and transmission spectroscopy. For the other two sulfurized films, the SnS_2_ secondary phase is observed in both samples in the Raman analysis. The Cu_2_S (PLD)/sulfurized sample displays a wider band gap (1.70 eV) than the two other films, which have an optimal E_g_ (between 1.40 and 1.56 eV) for use as an absorber layer in CZTS-based solar cell applications. In fact, ZnS being deposited by PLD limits Zn loss during deposition and annealing and helps preserve an important amount of the ZnS phase required for the CZTS phase formation. In summary, the MSPLD hybrid technique is beneficial in obtaining high-quality CZTS thin films for use as absorber layers in solar cells.

## Figures and Tables

**Table 1:** Position of the total reflection edge (2θ_TR_) and the average thickness (h_m_) (as well as their relative variation) of the as-deposited, annealed, and sulfurized CZTS thin films.

	2θ_TR_; Δρ_m_/ρ_o_			h_m_ (nm); Δh_m_/h_o_		
Sample	As-Deposited	Annealed @ 500 °C	Sulfurized @ 500 °C	As-Deposited	Annealed @ 500 °C	Sulfurized @ 500 °C
ZnS (PLD)	0.572°	0.566°; −2.1%	0.579; +2.5%	145	142; −2.0%	-
SnS_2_ (PLD)	0.589°	0.544°; −14.7%	0.565; +8.0%	147	126; −14.3%	-
Cu_2_S (PLD)	0.588°	0.567°; −7.0%	0.578; +3.4%	108	97; −10.2%	-

## Data Availability

The data presented in this study are available on a reasonable request from the corresponding author.

## References

[1] Ahmoum H., Boughrara M., Su’ait M.S., Li G., Chopra S., Wang Q., Kerouad M. (2020). Understanding the Effect of the Carbon on the Photovoltaic Properties of the Cu_2_ZnSnS_4_. Mater. Chem. Phys..

[2] Ataollahi N., Bazerla F., Malerba C., Chiappini A., Ferrari M., Di Maggio R., Scardi P. (2019). Synthesis and Post-Annealing of Cu_2_ZnSnS_4_ Absorber Layers Based on Oleylamine/1-Dodecanethiol. Materials.

[3] Sharmin A., Bashar M.S., Sultana M., Al Mamun S.M.M. (2020). Sputtered Single-Phase Kesterite Cu_2_ZnSnS_4_ (CZTS) Thin Film for Photovoltaic Applications: Post Annealing Parameter Optimization and Property Analysis. AIP Adv..

[4] Song X., Ji X., Li M., Lin W., Luo X., Zhang H. (2014). A Review on Development Prospect of CZTS Based Thin Film Solar Cells. Int. J. Photoenergy.

[5] Vanalakar S.A., Agawane G.L., Shin S.W., Suryawanshi M.P., Gurav K.V., Jeon K.S., Patil P.S., Jeong C.W., Kim J.Y., Kim J.H. (2015). A Review on Pulsed Laser Deposited CZTS Thin Films for Solar Cell Applications. J. Alloys Compd..

[6] Zhu Y., Chen Y., Shen T., Yi J., Gan G., Huang Q. (2017). Direct Current Magnetron Sputtered Cu_2_ZnSnS_4_ Thin Films Using a Ceramic Quaternary Target. J. Alloys Compd..

[7] Whittles T.J. (2018). Electronic Characterisation of Earth-Abundant Sulphides for Solar Photovoltaics.

[8] Ogugua S.N., Ntwaeaborwa O.M., Swart H.C. (2020). Latest Development on Pulsed Laser Deposited Thin Films for Advanced Luminescence Applications. Coatings.

[9] Rath M., Varadarajan E., Natarajan V., Ramachandra Rao M.S. (2018). A Comparative Study on Macroscopic and Nanoscale Polarization Mapping on Large Area PLD Grown PZT Thin Films. Ceram. Int..

[10] Oulad Elhmaidi Z., Abd-Lefdil M., El Khakani M.A. (2020). Photoconversion Optimization of Pulsed-Laser-Deposited p-CZTS/n-Si-Nanowires Heterojunction-Based Photovoltaic Devices. Nanomaterials.

[11] Oulad Elhmaidi Z., Pandiyan R., Abd-Lefdil M., El Khakani M.A. Pulsed Laser Deposition of CZTS Thin Films, Their Thermal Annealing and Integration into n-Si/CZTS Photovoltaic Devices. Proceedings of the 2016 International Renewable and Sustainable Energy Conference (IRSEC) 2016.

[12] Cazzaniga A., Crovetto A., Yan C., Sun K., Hao X., Ramis Estelrich J., Canulescu S., Stamate E., Pryds N., Hansen O. (2017). Ultra-Thin Cu_2_ZnSnS_4_ Solar Cell by Pulsed Laser Deposition. Sol. Energy Mater. Sol. Cells.

[13] Ettlinger R.B., Crovetto A., Canulescu S., Cazzaniga A., Ravnkilde L., Youngman T., Hansen O., Pryds N., Schou J. (2016). Formation of Copper Tin Sulfide Films by Pulsed Laser Deposition at 248 and 355 Nm. Appl. Phys. A.

[14] Cazzaniga A., Ettlinger R.B., Canulescu S., Schou J., Pryds N. (2014). Nanosecond Laser Ablation and Deposition of Silver, Copper, Zinc and Tin. Appl. Phys. A.

[15] Noroozi M., Petruhins A., Greczynski G., Rosen J., Eklund P. (2020). Structural and Mechanical Properties of Amorphous AlMgB_14_ Thin Films Deposited by DC Magnetron Sputtering on Si, Al_2_O_3_ and MgO Substrates. Appl. Phys. A.

[16] Olgar M.A., Klaer J., Mainz R., Levcenco S., Just J., Bacaksiz E., Unold T. (2016). Effect of Precursor Stacking Order and Sulfurization Temperature on Compositional Homogeneity of CZTS Thin Films. Thin Solid Film..

[17] Jheng B.-T., Liu P.-T., Wu M.-C. (2014). A Promising Sputtering Route for Dense Cu_2_ZnSnS_4_ Absorber Films and Their Photovoltaic Performance. Sol. Energy Mater. Sol. Cells.

[18] Xie M., Zhuang D., Zhao M., Zhuang Z., Ouyang L., Li X., Song J. (2013). Preparation and Characterization of a Cu_2_ZnSnS_4_ Thin Films and Solar Cells Fabricated from Quaternary Cu-Zn-Sn-S Target. Int. J. Photoenergy.

[19] Olgar M.A., Klaer J., Mainz R., Ozyuzer L., Unold T. (2017). Cu_2_ZnSnS_4_-Based Thin Films and Solar Cells by Rapid Thermal Annealing Processing. Thin Solid Film..

[20] Behera N., Mohan D.B. (2020). The Phase Optimization, Optical and Electrical Properties of Kesterite Cu_2_ZnSnS_4_ Thin Film Prepared by Single Target RF Magnetron Sputtering Technique for Solar Cell Application. Mater. Res. Express.

[21] Rudisch K., Davydova A., Riekehr L., Adolfsson J., Quaglia Casal L., Platzer-Björkman C., Scragg J. (2020). Prospects for Defect Engineering in Cu_2_ZnSnS_4_ Solar Absorber Films. J. Mater. Chem. A.

[22] Rondiya S., Rokade A., Jadhavar A., Nair S., Chaudhari M., Kulkarni R., Mayabadi A., Funde A., Pathan H., Jadkar S. (2017). Effect of Calcination Temperature on the Properties of CZTS Absorber Layer Prepared by RF Sputtering for Solar Cell Applications. Mater. Renew. Sustain. Energy.

[23] Zhao H., Xie J., Mao A. (2019). Effects of Bottom Layer Sputtering Pressures and Annealing Temperatures on the Microstructures, Electrical and Optical Properties of Mo Bilayer Films Deposited by RF/DC Magnetron Sputtering. Appl. Sci..

[24] Khalkar A., Lim K.-S., Yu S.-M., Patole S.P., Yoo J.-B. (2014). Deposition of Cu_2_ZnSnS_4_ Thin Films by Magnetron Sputtering and Subsequent Sulphurization. Electron. Mater. Lett..

[25] Gudmundsson J.T. (2020). Physics and Technology of Magnetron Sputtering Discharges. Plasma Sources Sci. Technol..

[26] Alvarez R., Garcia-Valenzuela A., Lopez-Santos C., Ferrer F.J., Rico V., Guillen E., Alcon-Camas M., Escobar-Galindo R., Gonzalez-Elipe A.R., Palmero A. (2016). High-Rate Deposition of Stoichiometric Compounds by Reactive Magnetron Sputtering at Oblique Angles. Plasma Process. Polym..

[27] Benetti D., Nouar R., Nechache R., Pepin H., Sarkissian A., Rosei F., MacLeod J.M. (2017). Combined Magnetron Sputtering and Pulsed Laser Deposition of TiO_2_ and BFCO Thin Films. Sci. Rep..

[28] Gómez-Solano R.E., Arias-Cerón J.S., Ríos-Ramírez J.J., Ortega-López M. (2020). Synthesis and Study of Structure and Phase Composition in Cu_2–X_S, Sn_x_S_y_, ZnS, Cu_x_SnS_y_ and CuZnSnS Pellets. J. Mater. Sci. Mater. Electron..

[29] Al-Hadeethi Y., Mkawi E.M., Al-Hartomy O., Bekyarova E. (2021). Solvothermal Synthesis of Kesterite Cu_2_ZnSnS_4_ Nanocrystals: Influence of Glycine Complexing Agent Concentration on Properties. Ceram. Int..

[30] Simandan I.-D., Sava F., Buruiana A.-T., Galca A.-C., Becherescu N., Burducea I., Mihai C., Velea A. (2021). Influence of Deposition Method on the Structural and Optical Properties of Ge_2_Sb_2_Te_5_. Materials.

[31] Huang L., Li J., Wang S., Zhong L., Xiao X. (2020). Forming an Ultrathin SnS Layer on Cu 2 ZnSnS 4 Surface to Achieve Highly Efficient Solar Cells with Zn(O,S) Buffer. Solar RRL.

[32] Tao J., Liu J., He J., Zhang Z., Jiang J., Sun L., Yang P., Chu J. (2014). Synthesis and characterization of Cu_2_ZnSnS_4_ thin films by the sulfurization of co-electrodeposited Cu–Zn–Sn–S precursor layers for solar cell applications. RSC Adv..

[33] Engberg S., Symonowicz J., Schou J., Canulescu S., Jensen K.M.Ø. (2020). Characterization of Cu_2_ZnSnS_4_ Particles Obtained by the Hot-Injection Method. ACS Omega.

[34] Havryliuk Y., Valakh M.Y., Dzhagan V., Greshchuk O., Yukhymchuk V., Raevskaya A., Stroyuk O., Selyshchev O., Gaponik N., Zahn D.R.T. (2018). Raman characterization of Cu_2_ZnSnS_4_ nanocrystals: Phonon confinement effect and formation of Cu_x_S phases. RSC Adv..

[35] Lin J., Xu J., Yang Y. (2020). Effect of Sulfur Powder Mass on the Formation of MoS_2_ Interface Layer between Cu_2_ZnSnS_4_ Thin Film and Mo Foil. Superlattices Microstruct..

[36] Azmi S., Moujib A., Layachi O.A., Matei E., Galca A.C., Zaki M.Y., Secu M., Rusu M.I., Grigorescu C.E.A., Khoumri E.M. (2020). Towards Phase Pure Kesterite Cu_2_ZnSnS_4_ Absorber Layers Growth via Single Step Free Sulfurization Electrodeposition under a Fix Applied Potential on Mo Substrate. J. Alloys Compd..

[37] Dimitrievska M., Fairbrother A., Fontane X., Jawhari T., Izquierdo-Roca V., Saucedo E., Perez-Rodriguez A. (2014). Multiwavelength excitation Raman scattering study of polycrystalline kesterite Cu_2_ZnSnS_4_ thin films. Appl. Phys. Lett..

[38] Chaudhari J.J., Joshi U.S. (2018). Fabrication of high quality Cu_2_SnS_3_ thin film solar cell with 1.12% power conversion efficiency obtain by low cost environment friendly sol-gel technique. Mater. Res. Express.

[39] Kumar M., Dubey A., Adhikari N., Venkatesan S., Qiao Q. (2015). Strategic Review of Secondary Phases, Defects and Defect-Complexes in Kesterite CZTS–Se Solar Cells. Energy Environ. Sci..

[40] Tan J.M.R., Lee H., Pedireddy S., Baikie T., Ling X.Y., Wong L.H. (2014). Understanding the Synthetic Paterhway of a Single-Phase Quaternary Semiconductor Using Surface-Enhanced Raman Scattering: A case of Wurtzite Cu_2_ZnSnS_4_ Nanoparticles. J. Am. Chem. Soc..

[41] Ahmad R., Brandl M., Distaso M., Herre P., Spiecker E., Hock R., Peukert W. (2015). A comprehensive study on the mechanism behind formation and depletion of Cu_2_ZnSnS_4_ (CZTS) phases. Cryst. Eng. Comm..

[42] Isik M., Gullu H.H., Terlemezoglu M., Bayrakli Surucu O., Parlak M., Gasanly N.M. (2020). Investigation of Band Gap Energy Versus Temperature for SnS_2_ thin films grown by RF-Magnetron Sputtering. Phys. B.

[43] Dimitrievska M., Boero F., Litvinchuk A.P., Delsante S., Borzone G., Perez-Rodriguez A., Izquierdo-Roca V. (2017). Structural Polymorphism in “Kesterite” Cu_2_ZnSnS_4_: Raman Spectroscopy and First-Principles Calculations Analysis. Inorg. Chem..

[44] Guc M., Levcenko S., Bodnar I.V., Izquierdo-Roca V., Fontane X., Volkova L.V., Arushanov E., Perez-Rodriguez A. (2016). Polarized Raman Scattering Study of Kesterite Type Cu_2_ZnSnS_4_ Single Crystals. Sci. Rep..

[45] Sava F., Diagne O., Galca A.-C., Simandan I.-D., Matei E., Burdusel M., Becherescu N., Becherescu V., Mihai C., Velea A. (2020). Secondary Crystalline Phases Influence on Optical Properties in Off-Stoichiometric Cu_2_S–ZnS–SnS_2_ Thin Films. Materials.

[46] Chen R., Fan J., Li H., Liu C., Mai Y. (2018). Efficiency Enhancement of Cu_2_ZnSnS_4_ Solar Cells via Surface Treatment Engineering. R Soc. Open Sci..

[47] Wang W., Chen G., Cai H., Chen B., Yao L., Yang M., Chen S., Huang Z. (2018). The Effects of SnS_2_ Secondary Phases on Cu_2_ZnSnS_4_ Solar Cells: A Promising Mechanical Exfoliation Method for Its Removal. J. Mater. Chem. A.

[48] Zaki M.Y., Nouneh K., Ebn Touhami M., Belakhmima R.A., Galca A.C., Pintilie L., Enculescu M., Baibarac M., Taibi M. (2018). Effect of Mixing Complexing Agents on the Properties of Electrodeposited CZTS Thin Films. Opt. Mater..

[49] Wang H., Yasin A., Quitoriano N.J., Demopoulos G.P. (2019). Aqueous-based Binary Sulfide Nanoparticle Inks for Cu_2_ZnSnS_4_ Thin Films Stabilized with Tin(IV) Chalcogenide Complexes. Nanomaterials.

[50] El Khouja O., Galca A.C., Nouneh K., Zaki M.Y., Ebn Touhami M., Taibi M., Matei E., Negrila C.C., Enculescu M., Pintilie L. (2021). Structural, Morphological and Optical Properties of Cu–Fe–Sn–S Thin Films Prepared by Electrodeposition at Fixed Applied Potential. Thin Solid Film..

[51] Malerba C., Biccari F., Azanza Ricardo C.L., Valentini M., Chierchia R., Müller M., Santoni A., Esposito E., Mangiapane P., Scardi P. (2014). CZTS Stoichiometry Effects on the Band Gap Energy. J. Alloys Compd..

[52] Hamanaka Y., Oyaizu W., Kawase M., Kuzuya T. (2017). Synthesis of Highly Non-Stoichiometric Cu_2_ZnSnS_4_ Nanoparticles with Tunable Bandgaps. J. Nanopart Res..

[53] Elhmaidi Z.O., Pandiyan R., Abd-Lefdil M., Saucedo E., El Khakani M.A. (2020). In-Situ Tuning of the Zinc Content of Pulsed-Laser-Deposited CZTS Films and Its Effect on the Photoconversion Efficiency of p-CZTS/n-Si Heterojunction Photovoltaic Devices. Appl. Surf. Sci..

[54] Aldalbahi A., Mkawi E.M., Ibrahim K., Farrukh M.A. (2016). Effect of Sulfurization Time on the Properties of Copper Zinc Tin Sulfide Thin Films Grown by Electrochemical Deposition. Sci. Rep..

[55] Zhang X., Fu E., Wang Y., Zhang C. (2019). Fabrication of Cu_2_ZnSnS_4_ (CZTS) Nanoparticle Inks for Growth of CZTS Films for Solar Cells. Nanomaterials.

[56] Chawla V., Clemens B. Effect of composition on high efficiency CZTSSe devices fabricated using co-sputtering of compound targets. Proceedings of the 2012 38th IEEE Photovoltaic Specialists Conference.

[57] Hlaing Oo W., Johnson J., Bhatia A., Lund E.-A., Nowell M.-M., Scarpulla M.-A. (2011). Grain Size and Texture of Cu_2_ZnSnS_4_ Thin Films Synthesized by Cosputtering Binary Sulfides and Annealing: Effects of Processing Conditions and Sodium. J. Electron. Mater..

[58] Bao W., Ichimura M. (2015). Influence of Secondary Phases in Kesterite-Cu_2_ZnSnS_4_ Absorber Material Based on the First Principles Calculation. Int. J. Photoenergy.

[59] Liu W.-S., Chen S.-Y., Huang C.-S., Lee M.-Y., Kuo H.-C. (2020). Investigation of Zn/Sn ratio for improving the material quality of CZTS thin films with the reduction of Cu_2-x_S secondary phase. J. Alloys Compd..

[60] Muhunthan N., Singh O.P., Singh S., Singh V.N. (2013). Growth of CZTS Thin Films by Cosputtering of Metal Targets and Sulfurization in H_2_S. Int. J. Photoenergy.

